# Musical experience modulates categorical perception of lexical tones in native Chinese speakers

**DOI:** 10.3389/fpsyg.2015.00436

**Published:** 2015-04-13

**Authors:** Han Wu, Xiaohui Ma, Linjun Zhang, Youyi Liu, Yang Zhang, Hua Shu

**Affiliations:** ^1^State Key Laboratory of Cognitive Neuroscience and Learning, Beijing Normal UniversityBeijing, China; ^2^Faculty of Linguistic Sciences, Beijing Language and Culture UniversityBeijing, China; ^3^Department of Speech-Language-Hearing Sciences and Center for Neurobehavioral Development, University of MinnesotaMinneapolis, MN, USA

**Keywords:** musical training, categorical perception, Chinese lexical tone, within-category discrimination, between-category discrimination

## Abstract

Although musical training has been shown to facilitate both native and non-native phonetic perception, it remains unclear whether and how musical experience affects native speakers’ categorical perception (CP) of speech at the suprasegmental level. Using both identification and discrimination tasks, this study compared Chinese-speaking musicians and non-musicians in their CP of a lexical tone continuum (from the high level tone, Tone1 to the high falling tone, Tone4). While the identification functions showed similar steepness and boundary location between the two subject groups, the discrimination results revealed superior performance in the musicians for discriminating within-category stimuli pairs but not for between-category stimuli. These findings suggest that musical training can enhance sensitivity to subtle pitch differences between within-category sounds in the presence of robust mental representations in service of CP of lexical tonal contrasts.

## Introduction

Categorical perception (CP), which refers to the phenomenon that gradually morphed sounds in a stimulus continuum tend to be perceived as discrete representations, has been studied for more than 50 years. While most of the early CP studies focused on segmental features (consonants and vowels; Liberman et al., 1957, 1961; Fry et al., 1962), there has been a recent surge of interests in suprasegmental features such as vowel duration contrasts of quantity languages (Nenonen et al., 2003; Ylinen et al., 2005) and lexical tone contrasts of tonal languages (Francis et al., 2003; Hallé et al., 2004; Xu et al., 2006; Xi et al., 2010). Evidence shows that the categorical nature of lexical tone perception depends on the pitch trajectory. For a continuum of flat tones ranging from one level to another, it is not categorically perceived (Francis et al., 2003), whereas a continuum involving contour tones is perceived categorically (Wang, 1976; Xu et al., 2006). In Mandarin Chinese there are four lexical tones, only one of which is a level tone (Howie, 1976). Thus, native perception of any Mandarin Chinese tonal continuum, due to the necessary involvement of contour tones, tends to be categorical with better sensitivity to between-category distinction relative to within-category differences. In recent years, much research interest has been oriented toward understanding the relationship between musical training and speech perception because music and speech share many acoustic commonalities as well as cognitive mechanisms. In fact, both signals convey information by means of timing, pitch, and timbre cues. Of these cues, pitch is of special interest because of its important roles in both domains. In music, two types of pitch information, i.e., contour and interval, are necessary to create melodies. Early studies have established that musicianship influences CP performance for pitch-related and duration-related auditory stimuli. For instance, the judgment of tonal intervals is shown to be more categorical in professionally trained musicians than non-musicians (Locke and Kellar, 1973; Siegel and Siegel, 1977; Burns and Ward, 1978). The evidence of categorical processing of musical pitch suggests that CP extends to signals other than speech and that it may be acquired from a special learning experience even when there are no natural sensory cues or physiologically built-in auditory discontinuities available to the listener (See Harnad, 1987 for discussions on theoretical explanations for CP). In speech, variations in pitch constitute essential prosodic patterns as associated with stress and intonational structure. Furthermore, in a tonal language like Chinese, pitch is also exploited to distinguish phonological contrasts at the syllable level. The advantage of musicianship in pitch processing has been confirmed by many previous studies. For example, compared to non-musicians, musicians are more skilled at learning to use non-native tonal contrasts to distinguish word meanings (Wong and Perrachione, 2007), and they are also more sensitive to pitch rises on final words of utterances irrespective of whether such changes occurred in their native language (Magne et al., 2006) or in a foreign language (Marques et al., 2007). Moreover, musicians are superior to non-musicians in discriminating subtle differences (Marie et al., 2012) and tracking accuracy (Wong et al., 2007) of pitch trajectories of both speech and non-speech sounds. In a recent study, it was reported that musicians showed greater accuracy and reaction time consistency than non-musicians for all types of stimuli in a discrimination task involving lexical tones, low-pass filtered speech tones, and violin sounds that carry the pitch contrasts of lexical tones (Burnham et al., 2014).

While the previous studies provide evidence for the transfer effects of long-term musical training on pitch processing of speech, it is theoretically important to investigate the extent of overlapping or domain specificity regarding the enhancement effects to better understand the perceptual and cognitive mechanisms involved in language and music processing Auditory cognitive neuroscience models for language processing all posit that speech perception involves multiple neural representations along the auditory pathway with dedicated low- and high-level neural structures performing acoustic analysis and phonological processing, respectively, that can be shaped and reshaped by learning experience (Hickok and Poeppel, 2007; Friederici, 2011). The study of lexical tone processing in the context of presence or absence of musical training for tonal-language speakers provides a great opportunity to explore the relationship between processing of speech and music. It remains unclear whether and how musical training affects CP of lexical tones by native tonal language speakers because the previous studies focused on pitch perception of musicians and non-musicians who are non-tonal language speakers. As a matter of fact, it is hotly debated whether the facilitatory effects of musical training on native language speech perception are attributed to musicians’ higher sensitivity to specific acoustic features or enhanced internal representation of phonological categories (Patel, 2011; Sadakata and Sekiyama, 2011; Marie et al., 2012; Kühnis et al., 2013; Bidelman et al., 2014). Previous research has provided robust evidence indicating that compared to non-musicians, musicians are more sensitive to various acoustic properties other than pitch (Milovanov et al., 2009; Marie et al., 2011, 2012; Sadakata and Sekiyama, 2011; Kühnis et al., 2013). However, whether the facilitatory effects extend to the additional higher-order phonological operation is not well known. CP of speech sounds arguably provides an optimal window for investigating whether and how musical training affects acoustic and/or linguistic processing of native language speech because both acoustic and phonological processing levels are involved in CP processes that perceptually evaluate carefully controlled within-category and between-category differences.

In the present study, identification and discrimination tasks were adopted to compare the performance of Chinese-speaking musicians and non-musicians in their CP of the lexical tone continuum from Tone1 to Tone4. Considering previous results reporting that musicians are superior in acoustic processing of pitch information, we predicted that musicians would be more accurate at discriminating within-category pairs. In terms of linguistic processing of between-category stimuli, we considered it an open-ended question whether musicians would have greater accuracies than non-musicians. Given the fact that CP of pitch direction by native Chinese speakers is also influenced by stimulus complexity (speech vs. non-speech; Xu et al., 2006), sine-wave tones re-synthesized from the Tone1 to Tone4 continuum were also included in order to explore domain specificity of speech processing and examine whether the stimulus complexity effect could also be modulated by musical experience.

## Materials and Methods

### Subjects

Sixty-four native speakers of Mandarin Chinese participated in the experiment: Thirty-two musicians (18 female, 14 male; mean age = 19.2, range 17-23) and 32 non-musicians (20 female, 12 male; mean age = 19.7, range 18-25). The musicians had undergone at least seven years of continuous formal western instrumental music training, and they had regular practice and current opportunities to play an instrument. The non-musicians were selected with the inclusion criterion that they had never received formal musical training within the last five years and less than 2 years of musical experience prior to that (Wong et al., 2007; Wayland et al., 2010). The two groups of subjects were, respectively, recruited from China Conservatory of Music and Beijing Normal University. They reported having no history of hearing impairment, neurological, psychiatric, or neuropsychological problems. All signed an informed consent in compliance with a protocol approved by the research ethics committee of Beijing Normal University and were paid for their participation.

### Stimuli

**FIGURE 1:**
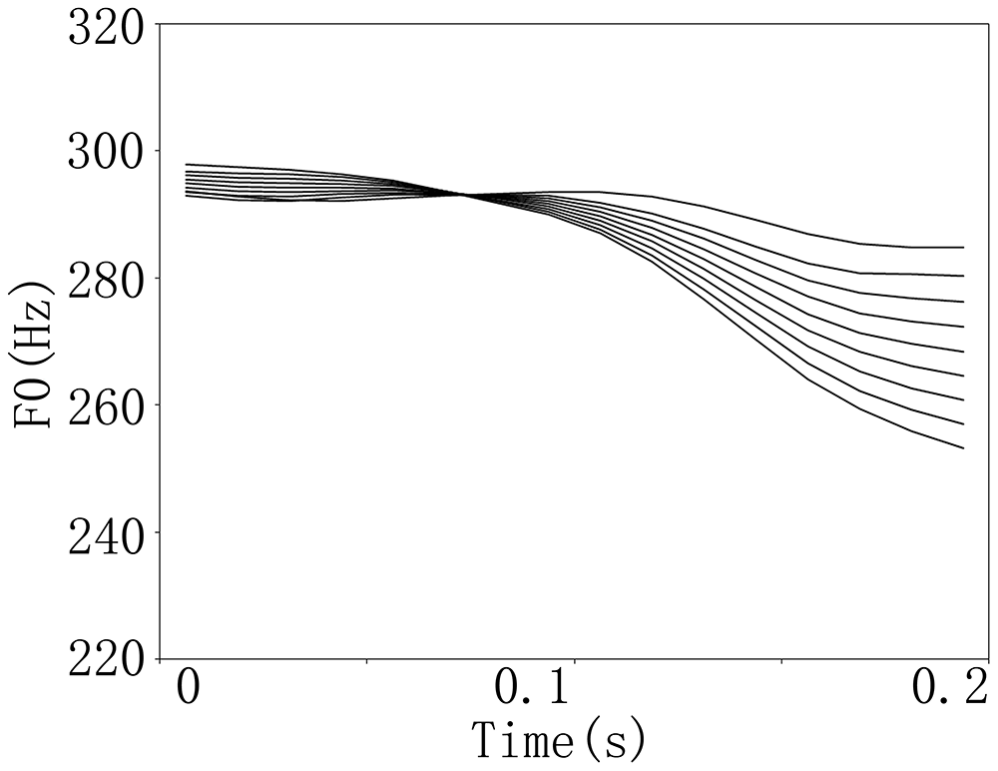
**Tone contours of the continuum from /ba1/ to /ba4/**.

There were two sets of continua for the speech and non-speech stimuli (**Figure 1**). The speech stimuli were the Chinese monosyllables /pa/ that differed in their lexical tones (i.e., the high level tone, Tone1, and the high falling tone, Tone4). The original stimuli were first recorded at a sampling rate of 44.1 kHz from a female native Chinese speaker. The syllables were then digitally edited to have a duration of 200 ms using Sound-Forge (SoundForge9, Sony Corporation, Japan). In order to isolate the lexical tones and keep the rest of the acoustic features equivalent, pitch tier transfer was performed using the Praat software (http://www.fon.hum.uva.nl/praat/). This procedure generated two stimuli, /pa1/ and /pa4/, which were identical with each other except for the pitch contour difference. The /pa1/ and /pa4/ stimuli were then taken as the endpoint stimuli to create a lexical tone continuum. A morphing technique was performed in Matlab (Mathworks Corporation, USA) using STRAIGHT (Kawahara et al., 1999) in eight equal intervals. The non-speech stimuli were sine-wave tones with the same pitch contours as the speech stimuli. All stimuli were normalized in RMS intensity.

### Procedures

Categorical perception of the speech and non-speech continua was examined in discrimination and identification tasks. In order to avoid the possible effects that perception of one stimulus type might have on the other due to familiarity with the experimental procedure, we included a 6-month interval between speech and non-speech tests. Furthermore, the use of discrimination/identification tasks and speech/non-speech stimuli were counterbalanced among the participants in each subject group to control effects due to stimulus presentation sequence.

In the identification task, participants listened to stimuli from the speech/non-speech continuum presented in isolation. They were instructed to press the “F” key upon hearing a “level” pitch, or the “J” key on a computer keyboard upon hearing a “falling” pitch. There were six occurrences of each of the nine stimuli (54 trials) presented in random order. The rate of presentation was self-paced with 1-s pause after response.

In the discrimination task, two stimuli were presented consecutively with a 500-ms interstimulus interval (ISI) and participants were instructed to judge whether the two sounds were identical or not by pressing the “1” (same) or 2 (different) key on the keyboard. There were three occurrences of each of the fourteen 2-step pairs (42 trials) randomly presented, either in forward (1–3, 2–4, 3–5, 4–6, 5–7, 6–8, 7–9) or reverse order (3–1, 4–2, 5–3, 6–4, 7–5, 8–6, 9–7). Foil trials involving identical stimuli in the pairwise presentation were also included. The rate of presentation was self-paced as in the identification task.

### Data Analysis

To investigate the effects of group (musician vs. non-musician) and stimulus type (speech vs. non-speech) on identification and discrimination performance, five essential characteristics of CP, i.e., sharpness and location of category boundary, between- and within-category discrimination accuracy, and peakedness of discrimination function were calculated (Xu et al., 2006).

Logistic regression between identification score and step number was used to obtain the identification function. The Generalized Estimating Equations estimated regression coefficient b1 was used to evaluate the slope of the fitted logistic curve which is an indication of the sharpness of the categorical boundary (Liang and Zeger, 1986). The categorical boundary location was derived from the value of step number (*X*_cb_) corresponding to the 50% identification score.

The obtained discrimination data were examined by three different measures: between-category discrimination accuracy (*P*_bc_), which was measured from the comparison unit corresponding to the categorical boundary (*X*_cb_) determined from the subgroup identification functions (*P*_bc_ = *P*_46_); within-category discrimination accuracy (*P*_wc_), which was the average of the two comparison units at the ends of the continuum (*P*_13_ and *P*_79_); and peakedness of the discrimination function (*P*_pk_), estimated by *P*_bc_ minus *P*_wc_. All the discrimination measures were obtained by computing the proportion of “different” responses.

## Results

**FIGURE 2:**
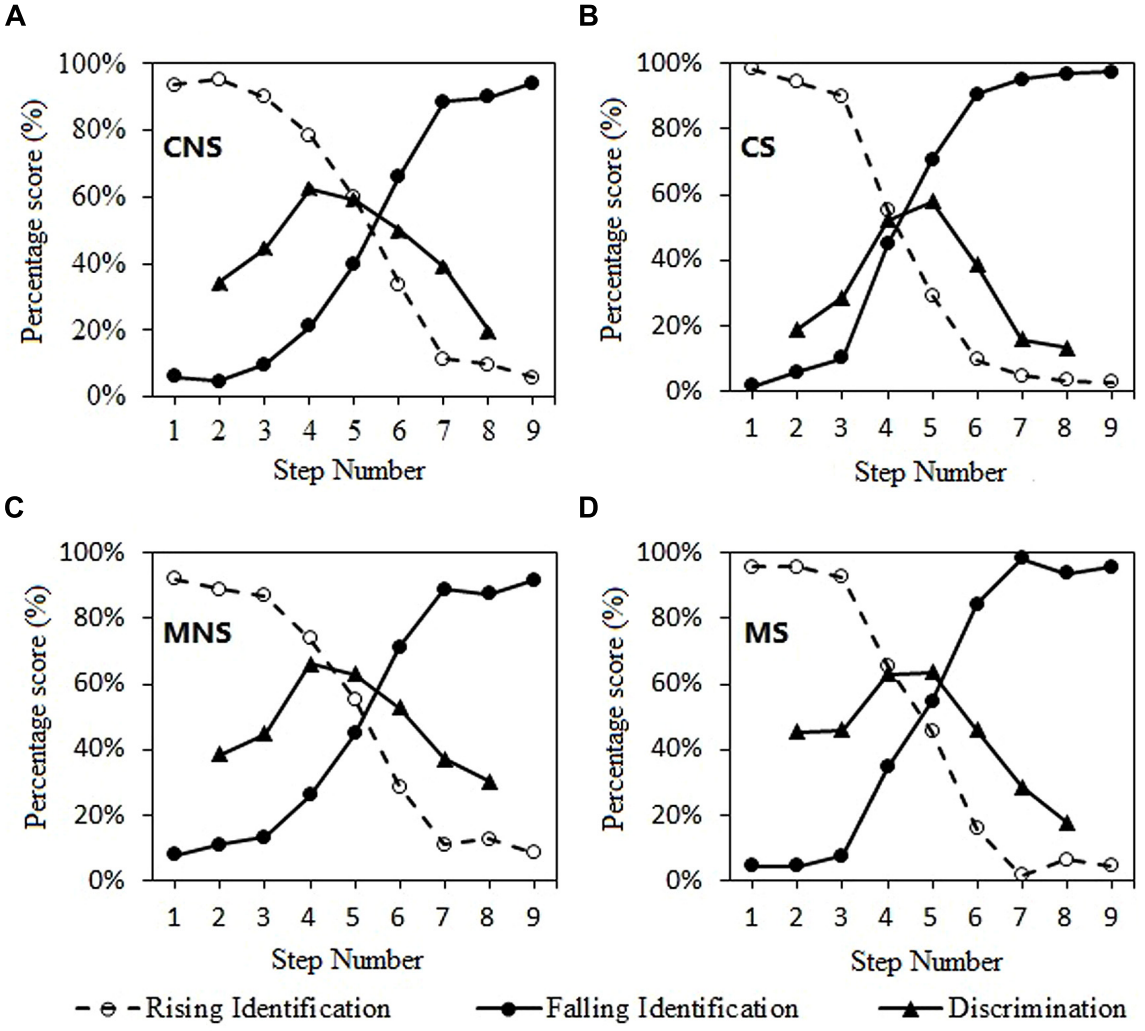
**Logistic identification functions and two-step discrimination curves**. The “level” logistic response functions were plotted by reflecting the “falling” logistic response functions (Plevel = 1-Pfalling). The discrimination curves were obtained from proportion of “different responses.” **(A)** non-musician, non-speech; **(B)** non-musician, speech; **(C)** musician, non-speech; **(D)** musician, speech.

**Table 1** shows the estimated regression coefficients for the mean logistic response functions. **Table 2** shows the *d*-prime measures for discrimination of each subject group and stimulus type. The identification and discrimination curves are depicted in **Figure 2**.

**Table 1:** GEE estimates of regression coefficients (*b*_0_, *b*_1_) and the derived categorical boundary (*X*_cb_) for each subgroup (Musician and Non-musician Groups; Speech and Non-speech conditions).

Subgroup	*b*_0_	*b*_1_	*X*_cb_ = -*b*_0_/*b*_1_
MusicianSP	6.8277	-1.4527	4.7216
MusicianNonSP	6.2251	-1.2193	5.0771
Non-musicianSP	7.3825	-1.7135	4.3619
Non-musicanNonSP	7.3710	-1.3778	5.2870

**Table 2:** *d*-prime measures for discrimination of each subgroup (Musician and Non-musician Groups; Speech and Non-speech conditions).

Subgroup	*P*_bc_ *d*-prime	*P*_wc_ *d*-prime
MusicianSP	1.60 (0.27)	0.50 (0.14)
MusicianNonSP	1.59 (0.22)	0.61 (0.18)
Non-musicianSP	1.68 (0.24)	0.09 (0.18)
Non-musicianNonSP	1.77 (0.19)	0.67 (0.14)

Each of the five measures was analyzed by a two-way ANOVA model for group (musician vs. non-musician) and stimulus type (speech vs. non-speech) effects. The steepness of the category boundary (b1) had a significant main effect of stimulus type [*F*(1,62) = 6.575, *p* = 0.013], indicating that CP of the speech continuum is stronger than non-speech. There was a significant main effect of stimulus type [*F*(1,62) = 25.264, *p* < 0.001] and group × stimulus type interaction effect [*F*(1,62) = 4.997, *p* = 0.029] for the location of category boundary (*X*_cb_). The category boundary of the speech continuum shifted to the high level end compared with the non-speech continuum for both groups. This boundary shift phenomenon was more obvious in the non-musician group.

**FIGURE 3:**
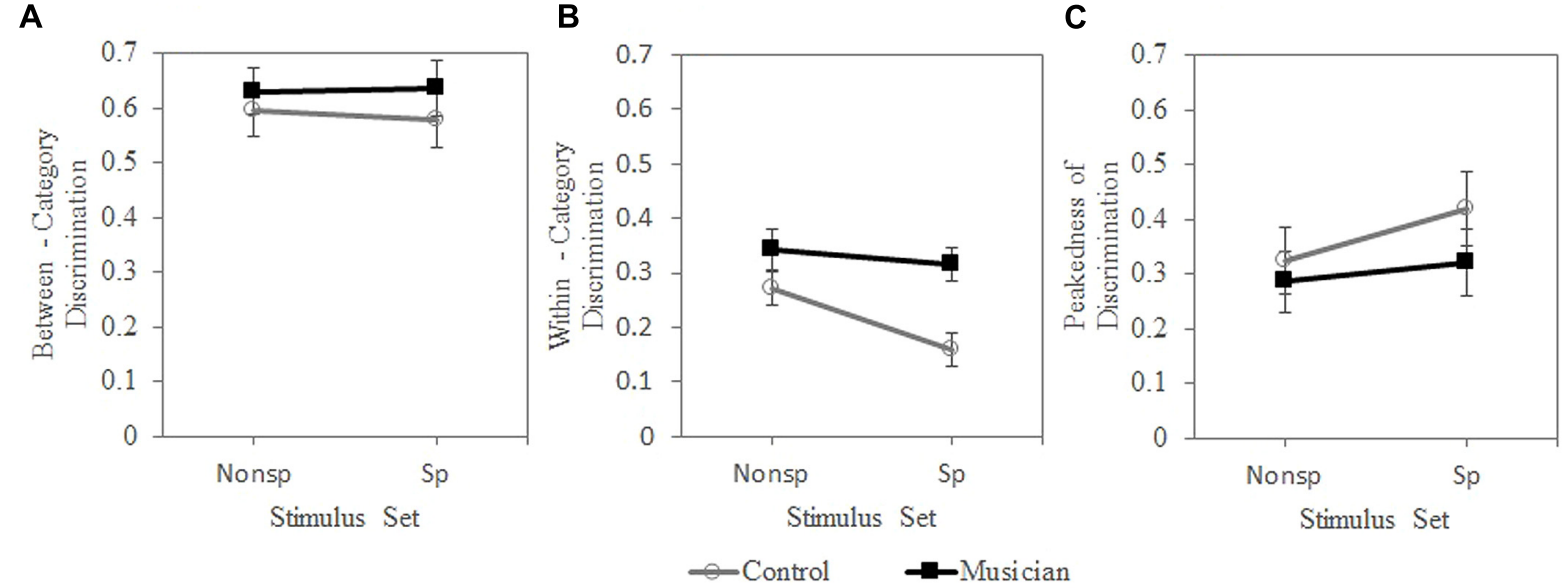
**Proportion of “different responses for the discrimination task.” **(A)** between-category, **(B)** within-category, **(C)** peakedness of discrimination**. S, speech; NS, non-speech.

The within-category discrimination accuracy (*P*_wc_) had significant main effects of subject group [*F*(1,62) = 11.596, *p* = 0.001] and stimulus type [*F*(1,62) = 5.227, *p* = 0.026]. The group × stimulus type interaction effect was not significant, indicating that musicians were superior to non-musicians in discriminating the within-category pitch difference in both the speech and non-speech continua. For both groups, non-speech yielded better within-category discrimination accuracy than speech. For both the between-category discrimination accuracy (*P*_bc_) and peakedness of the discrimination function (*P*_pk_), there was no significant main effect or interaction (**Figure 3**).

## Discussion

While previous work has shown that musical expertise enhances the ability to categorize linguistically relevant sounds of a second language (Wong and Perrachione, 2007; Sadakata and Sekiyama, 2011; Mok and Zuo, 2012), and to discriminate some important acoustic cues of both speech and non-speech sounds (Magne et al., 2006; Marques et al., 2007; Marie et al., 2012; Kühnis et al., 2013), the current results link and extend these results by demonstrating similarities as well as differences in how Chinese musicians and non-musicians perform in the CP of native tonal contrasts. Our identification results showed that it was more obvious in the non-musician group that category boundary of the speech continuum shifted to the high level end compared with the non-speech continuum. Nevertheless, at the subject group level, musicians and non-musicians did not differ in either the locations or sharpness of the category boundaries of both continua. The identification results were further confirmed by the discrimination findings showing that the two groups had similar between-category discrimination accuracy and peakedness of the discrimination function. However, the musicians had higher within-category discrimination accuracy irrespective of the stimulus type. Taken together, these results suggest that musical training affects native Chinese speakers’ CP of lexical tones resulting from differential perceptual weighting for the acoustic and linguistic dimensions of processing.

Positive transfer effects of musical training to various cognitive abilities have been confirmed by many previous studies. For example, musical training is associated with an increase in general IQ (Schellenberg, 2006) as well as enhanced working memory (Tierney et al., 2008). Various linguistic abilities such as verbal memory (Ho et al., 2003) and reading (Anvari et al., 2002) have also been shown to correlate with musical skills. However, at the more fundamental level of speech perception, whether musical training enhances the ability to categorize the speech sounds of native language is not well known. To our knowledge, this issue is directly examined only by two studies but the results are inconsistent with each other. Bidelman et al. (2014) found that musicians had a steeper category boundary than non-musicians in a vowel identification task, indicating that musicians have heightened internal representations of native phonological categories, whereas musicians’ advantage in identifying native vowels is not found in another study (Sadakata and Sekiyama, 2011). Using both identification and discrimination tasks, the current study showed that musicians and non-musicians did not differ in terms of linguistic operations for between-category identification and discrimination of native speech sounds. We argue that the phonetic inventories along with the phonetic boundaries in the speech continua for the native language were acquired and refined in early development (Zhang et al., 2005), i.e., long before the onset of music lessons, and thus more resistant to change brought about by musical experience. The result that musical training enhanced within-category discrimination accuracy is in accordance with a large number of previous findings that have consistently demonstrated musicians are typically more sensitive to various acoustic properties, in particular, pitch information of pure tones, music, and speech sounds (Wong et al., 2007; Milovanov et al., 2009; Marie et al., 2011, 2012; Sadakata and Sekiyama, 2011; Kühnis et al., 2013). Together with previous findings, our identification and discrimination results seem to indicate that musical training strongly enhances the sensitivity to subtle acoustic difference between within-category sounds, while the perceptual space between phonological contrasts of native language is more robust and less likely to be affected.

Neuroimaging studies have revealed hierarchical brain processing that operates on the flow of information from acoustic to phonological facets of the speech network, with the upstream areas (e.g., Heschl’s gyrus and the superior temporal gyrus) performing initial acoustic analysis and the downstream regions (e.g., the superior temporal sulcus and middle temporal gyrus) performing higher-level phonological processing (Wessinger et al., 2001; Joanisse et al., 2007; Okada et al., 2010; Zhang et al., 2011). The impact of intensive musical training on auditory processing has been well documented, however, functional and structural changes associated with musical training mainly take place in the upstream brain areas, especially Heschl’s gyrus and the planum temporale rather than the downstream regions (Pantev et al., 2001; Hyde et al., 2009; Moreno et al., 2009; Elmer et al., 2012). In this regard, our behavioral results are also consistent with the neuroimaging findings.

Finally, our study revealed an effect of stimulus type (speech vs. non-speech) in both identification and discrimination tasks. Compared with speech, perception of the non-speech continuum had a shallower category boundary, higher within-category discrimination accuracy and shifted the boundary location toward the high falling end of the continuum. These results are consistent with an earlier study (Xu et al., 2006) adopting the same tasks and similar stimuli, indicating that stimulus complexity affects the CP of pitch direction of native Chinese speakers. More importantly, only the boundary location showed an interaction effect between stimulus type and group, i.e., different boundary locations between the speech and non-speech continua were only observed in the non-musician group. Such results indicate that musical experience contributes to the approximation of the category boundaries of the speech/non-speech continua, likely by improved fine-graded auditory skills in the professional musicians.
